# Potential clinical utility of liquid biopsy in early-stage non-small cell lung cancer

**DOI:** 10.1186/s12916-022-02681-x

**Published:** 2022-12-14

**Authors:** Haifeng Shen, Yichen Jin, Heng Zhao, Manqi Wu, Kai Zhang, Zihan Wei, Xin Wang, Ziyang Wang, Yun Li, Fan Yang, Jun Wang, Kezhong Chen

**Affiliations:** Thoracic Oncology Institute, Department of Thoracic Surgery, Peking University People’s Hospital, Peking University, Xi Zhi Men South Ave No.11, Beijing, 100044 China

**Keywords:** Early-stage NSCLC, Liquid biopsy, Diagnostic accuracy, Prognostic values, Molecular residual disease, Predictive values, Treatment evaluation

## Abstract

**Background:**

Liquid biopsy has been widely researched for early diagnosis, prognostication and disease monitoring in lung cancer, but there is a need to investigate its clinical utility for early-stage non-small cell lung cancer (NSCLC).

**Methods:**

We performed a meta-analysis and systematic review to evaluate diagnostic and prognostic values of liquid biopsy for early-stage NSCLC, regarding the common biomarkers, circulating tumor cells, circulating tumor *DNA* (*ctDNA*), methylation signatures, and *microRNAs*. Cochrane Library, PubMed, EMBASE databases, ClinicalTrials.gov, and reference lists were searched for eligible studies since inception to 17 May 2022. Sensitivity, specificity and area under the curve (AUC) were assessed for diagnostic values. Hazard ratio (HR) with a 95% confidence interval (CI) was extracted from the recurrence-free survival (RFS) and overall survival (OS) plots for prognostic analysis. Also, potential predictive values and treatment response evaluation were further investigated.

**Results:**

In this meta-analysis, there were 34 studies eligible for diagnostic assessment and 21 for prognostic analysis. The estimated diagnostic values of biomarkers for early-stage NSCLC with AUCs ranged from 0.84 to 0.87. The factors TNM stage I, T1 stage, N0 stage, adenocarcinoma, young age, and nonsmoking contributed to a lower tumor burden, with a median cell-free *DNA* concentration of 8.64 ng/ml. For prognostic analysis, the presence of molecular residual disease (*MRD*) detection was a strong predictor of disease relapse (RFS, HR, 4.95; 95% CI, 3.06–8.02; *p* < 0.001) and inferior OS (HR, 3.93; 95% CI, 1.97–7.83; *p* < 0.001), with average lead time of 179 ± 74 days between molecular recurrence and radiographic progression. Predictive values analysis showed adjuvant therapy significantly benefited the RFS of *MRD* + patients (HR, 0.27; *p* < 0.001), while an opposite tendency was detected for *MRD − *patients (HR, 1.51; *p* = 0.19). For treatment response evaluation, a strong correlation between pathological response and *ctDNA* clearance was detected, and both were associated with longer survival after neoadjuvant therapy.

**Conclusions:**

In conclusion, our study indicated liquid biopsy could reliably facilitate more precision and effective management of early-stage NSCLC. Improvement of liquid biopsy techniques and detection approaches and platforms is still needed, and higher-quality trials are required to provide more rigorous evidence prior to their routine clinical application.

**Supplementary Information:**

The online version contains supplementary material available at 10.1186/s12916-022-02681-x.

## Background

Lung cancer was the second most commonly diagnosed cancer and the leading cause of cancer death in 2020, with an estimated 2.2 million new cancer cases and 1.8 million deaths worldwide [1]. Approximately 60% of lung cancer patients have distant metastasis at the initial diagnosis, and a substantial number of patients still progress to local recurrence or distant metastasis after curative-intent treatment [2, 3]. Low-dose computed tomography (LDCT) has been suggested for lung cancer screening, with a significant relative reduction in mortality for lung cancer patients [4]. However, distinguishing small malignant nodules in LDCT from benign lesions is particularly challenging due to the high false-positive rate [5–7]. In practice, a significant proportion of patients still suffer from ambiguous disease progression during radiological follow-up after radical surgery. Presently, the prognostic stratification of non-small cell lung cancer (NSCLC) using factors such as tumor, node, metastasis (TNM) classification, airway spread, and pathological subtype has shown limited effectiveness, with some low-risk patients experiencing postoperative relapse [8]. Therefore, more effective approaches and biomarkers will contribute to early detection, precision medicine, individualized treatment, and prognostication of lung cancer, and they are urgently needed.

Recently, with the advantages of noninvasiveness, ease of access, reproducibility, good reflection of the overall state of the tumor, and real-time surveillance, liquid biopsy has been widely researched and has shown promising efficacy in early diagnosis, prognostication, and disease monitoring in lung cancer. Circulating tumor cells (CTCs), circulating tumor *DNA (ctDNA)*, methylation signatures, and *microRNAs* are the most commonly detected biomarkers [9, 10]. The clinical applications of liquid biopsy have been gradually established for advanced-stage NSCLC and metastatic disease. With the development of molecular biological detection technologies and platforms, combined with multianalytical approaches and machine learning models, the clinical performance of liquid biopsy in detecting diagnostic, prognostic, and predictive biomarkers for early-stage NSCLC has been further investigated in the last few decades [11–16]. Here, we performed this systematic review and meta-analysis to discuss the advantages and current limitations of liquid biopsy in the management of localized NSCLC, regarding the commonly detected biomarkers, CTCs, *ctDNA*, methylation signatures, and *microRNAs*. The potential clinical utility of liquid biopsy in early-stage NSCLC diagnosis, prognosis, and clinical monitoring of treatment response or recurrence will also be explored.

## Methods

### Study selection

This study followed the Preferred Reporting Items for Systematic Reviews and Meta-analysis (PRISMA) 2020 statements checklist (Additional file 1: Table S1) [17]. Literature search was conducted in Cochrane Library, PubMed, EMBASE databases, ClinicalTrials.gov, and reference lists by 2 researchers (H.S. and Y.J.) independently. Studies published since inception to 17 May 2022 were included. To perform a comprehensive search, we used the following keywords and MeSH terms in different patterns: (“*Lung Neoplasm*”) AND (“*Liquid Biopsy*” OR “*Circulating Tumor Cell*” OR “*Circulating Tumor DNA*” OR “*DNA Methylation*” OR “*Circulating Tumor RNA*”) (Additional file 2: Table S1).

### Eligibility criteria

The following criteria were used for study inclusion: studies that evaluated the diagnostic and prognostic values of liquid biopsy for early-stage NSCLC, regarding the common biomarkers, CTCs, *ctDNA*, methylation signatures, and *microRNAs*; adequate data to construct the diagnostic 2 × 2 table for diagnostic assessment; sufficient survival data to obtain the hazard ratios (HRs) for the prognostic analysis in the preoperative, postoperative, and postchemotherapy time point; and the most recent or completed study if based on overlapping patients. The exclusion criteria were as follows: studies without any relevant data for analysis; stage IV or advanced-stage NSCLC; the involved sample size was fewer than 10; papers that were not published in English; and commentaries, editorials, reports, reviews, letters, and experiments.

### Data extraction

The following data were extracted in a standardized form: the publication details, study design, patient characteristics, stages, biopsy method, type of biomarker, the true positive, false positive, false negative, true negative for the analysis of the sensitivity and specificity. Additionally, the concentrations of circulating cell-free *DNA* (*cfDNA*) in preoperative plasma of eligible cohorts were also extracted for the exploration of related clinical factors, including sex, age, TNM stage, smoking, and histopathology. And if the studies reported the association between circulating biomarkers and short- and long-term outcomes of the NSCLC patients, follow-up duration and the hazard ratio (HR) with a 95% confidence interval (CI) were extracted from the regression or survival plot of recurrence-free survival (RFS) and overall survival (OS). The prognostic analysis for molecular residual disease (*MRD*) detection focused on the survival data at postoperative time point. The reported lead time of biomarker (e.g., *ctDNA*) detection preceding radiographic progression was also listed and summarized. As for predictive value analysis, original survival data were extracted from the Kaplan–Meier curves comparing RFS between *MRD/ctDNA*-positive patients receiving adjuvant therapy and not receiving adjuvant therapy and comparing RFS between *MRD/ctDNA*-negative patients receiving adjuvant therapy and not receiving adjuvant therapy. In terms of treatment response evaluation for neoadjuvant therapy, relevant data in *ctDNA* clearance and assessment of pathological response were collected and analyzed. Any discrepancies were assessed by a third author (K.C.).

### Risk of bias assessment

The quality assessment of the included studies was evaluated by the Quality Assessment for Studies of Diagnostic Accuracy Score-2 (QUADAS-2) tool [18], with 4 different domains: patient selection, index test, reference standard, and flow and timing. And for the cohort studies involved in prognostic analysis, the quality assessment was followed by the Newcastle–Ottawa Scale [19]. Publication bias was detected by Deeks’ funnel plot.

### Statistical analysis

Diagnostic meta-analysis was based on the MIDAS module (Stata module for meta-analytical integration of diagnostic test accuracy studies) [20] and bivariate approach [21]. The pooled sensitivity and specificity were calculated by the accuracy data, and the summary receiver operative curve (SROC) was generated by “mada” package, and the area under the curve (AUC) was evaluated. Analysis of factors related to preoperative *cfDNA* concentration was conducted by Kruskal–Wallis test. In terms of prognostic analysis, the RFS and OS were measured by HRs and 95% CIs that were directly reported in the included studies. Otherwise, survival data that were not presented numerically in articles were extracted from the Kaplan–Meier curve using Engauge Digitizer version 12, and the HRs were calculated by the Parmar and Tierney methods [22, 23]. The random-effects model was pooled due to the high heterogeneity of the studies (*p* < 0.10 or *I*^*2*^ > 50%). Otherwise, the fixed-effects model was used. Subgroup analysis was performed by the preoperative, postoperative, and postchemotherapy time point, while the prognostic analysis for *MRD* detection was based on the survival data at postoperative time point. The lead time analysis was estimated and summarized by the Wan and Luo methods [24, 25] and then pooled and presented in an estimated average using the ggplot2 package. The forest plots of predictive value analysis were pooled in *MRD/ctDNA*-positive group and *MRD/ctDNA*-negative group. Correlation between pathological response and *ctDNA* clearance was conducted by the 2-sided Fisher’s exact test [26]. Survival analysis in pathological response and *ctDNA* clearance was also pooled in a forest plot. Statistical analyses were performed by Stata 15.0 software (Stata Corporation, College Station, TX) and RStudio 4.1.3. Statistical significance was set at *p* < 0.05.

## Results

### Literature selection and study characteristics

A flow diagram of the literature search is shown in Additional file 3: Fig. S1. After exclusion of studies, a total of 53 articles were included in the meta-analysis with 34 studies [14, 16, 27–58] eligible for diagnostic assessment and 21 [12–15, 33, 59–74] for prognostic analysis. Summary characteristics of the included studies were demonstrated in Additional file 2: Table S2.

### Assessment of bias

Assessment of the study quality was evaluated using the QUADAS-2 tool (Additional file 2: Table S3) and Newcastle–Ottawa Scale (Additional file 2: Table S4). There was no significant publication bias determined by the Deeks’ funnel plot (*p* = 0.39, Additional file 3: Fig. S2) in diagnostic study nor by formal statistical tests of Egger’s test in prognostic analysis (Additional file 3: Fig. S3).

### Diagnostic performance

**Fig. 1:**
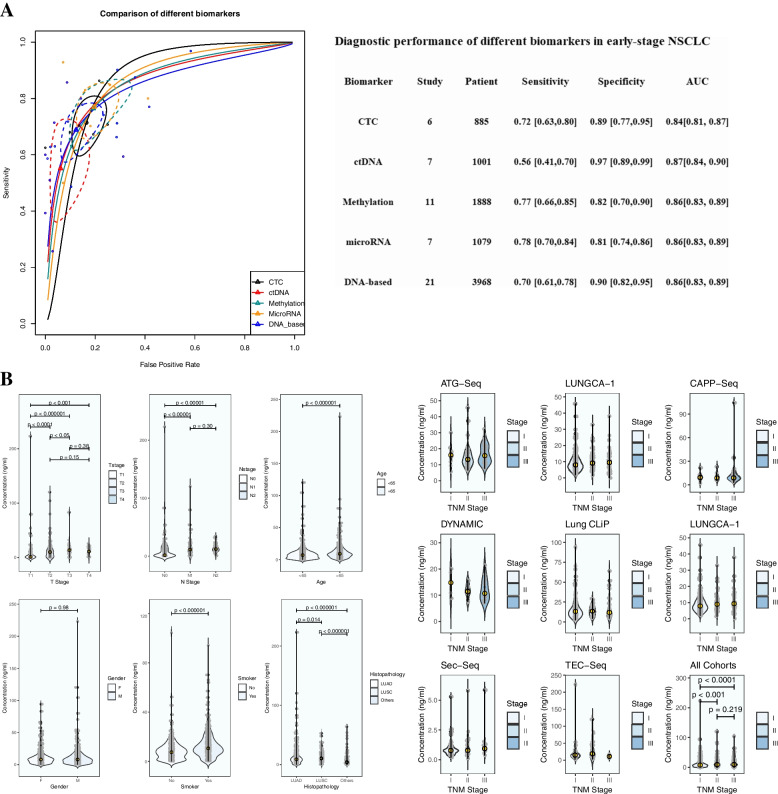
**A** Comparison of SROC of different biomarkers. **B** Analysis of cell-free *DNA* concentration

For the diagnostic analysis, 2917 healthy controls and 3015 patients with early-stage NSCLC were included, among whom 1537 patients were limited to stage I. The biomarkers included CTC in 6 eligible studies with 885 participants, *ctDNA* in 7 studies with 1001 participants, *DNA* methylation in 11 studies with 1888 participants, and *microRNAs* in 7 relevant articles with 1079 participants. The estimated diagnostic values of different common biomarkers for early-stage NSCLC with AUCs ranged from 0.84 to 0.87 (Fig. 1A). Additionally, the comparison of the diagnostic values between different biomarkers showed no significant differences, with a similar ROC-AUC and overlapping 95% confidence ellipses. In particular, a lower AUC was calculated when the analysis was limited to stage I disease (Additional file 2: Table S5).

### Analysis of the *cfDNA* concentration

Eight cohorts of 941 patients with early-stage NSCLC were reanalyzed [13–15, 35, 40, 42, 66, 69]. The relative factors included TNM stage (I, II, and III), T stage (T1, T2, T3, and T4), N stage (N0, N1, and N2), age (≥ 65 vs. < 65), sex, smoking status, and histopathology. The median *cfDNA* concentration of the preoperative plasma samples from all patients with stage I–III NSCLC was 8.64 ng/ml (Additional file 2: Table S6, original data in Additional file 4: Table S1). For TNM stage, the median *cfDNA* concentration in stage I was 7.58 ng/ml, significantly lower than the median concentrations in stage II (9.86 ng/ml, *p* < 0.001) and III (10.03 ng/ml, *p* < 0.0001). There was no significant difference between the median concentrations in stages II and III (*p* = 0.22) (Fig. 1B). The median *cfDNA* concentration in the T1 stage was 1.09 ng/ml, significantly lower than the median concentrations in the higher T stages. Similarly, the median *cfDNA* concentrations in N0 stage (1.70 ng/ml) were significantly lower than in N1 and N2 stages. Older patients had significantly higher *cfDNA* concentrations than younger patients (≥ 65 vs. < 65, 9.03 ng/ml vs. 6.99 ng/ml, *p* < 0.000001). Smoking was also associated with significantly higher *cfDNA* concentrations (smoking vs. nonsmoking, 10.64 ng/ml vs. 7.52 ng/ml, *p* < 0.000001). No significant difference was detected between the sexes (female vs. male, 7.83 ng/ml vs. 7.90 ng/ml, *p* = 0.98). For different histopathologies, the results showed that lung adenocarcinoma (LUAD) was associated with significantly lower *cfDNA* concentrations than lung squamous cell carcinoma (LUSC) (LUAD vs. LUSC, 9.03 ng/ml vs. 10.78 ng/ml, *p* = 0.014) (Fig. 1B).

### Prognostic performance

**Fig. 2:**
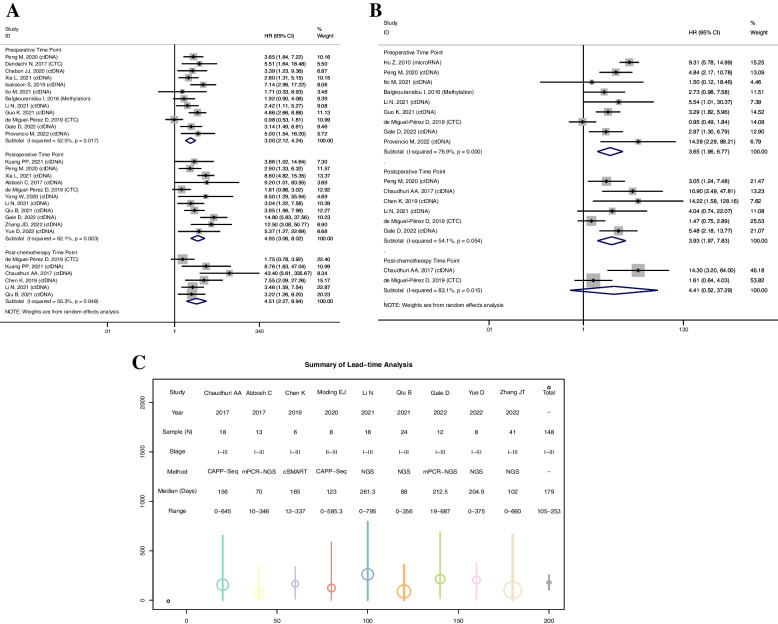
Forest plots of **A** recurrence-free survival and **B** overall survival. **C** Summary of lead time analysis

To evaluate the prognostic values of liquid biopsy in early-stage NSCLC, 21 eligible studies with 2143 patients were included [12–15, 33, 59–74]. Most of the studies referred to *ctDNA* biomarker, with only one relevant study focusing on *DNA* methylation [33], 2 regarding CTCs [60, 61], and 1 referring to *microRNAs* [59]. Subgroup analysis was performed by the preoperative, postoperative, and postchemotherapy time point, while the prognostic analysis for *MRD* detection was based on the survival data at postoperative time point. The forest plots showed that *MRD* detection after curative intent treatment was a strong predictor of disease relapse (RFS, HR, 4.95; 95% CI, 3.06–8.02; *p* < 0.001, Fig. 2A) and a shorter OS (HR, 3.93; 95% CI, 1.97–7.83; *p* < 0.001, Fig. 2B). Likewise, biomarkers positive in the preoperative blood samples were also associated with significantly inferior RFS (HR, 3.00; 95% CI, 2.12–4.24; *p* < 0.001, Fig. 2A) and OS (HR, 3.65; 95% CI, 1.96–6.77; *p* < 0.001, Fig. 2B). Similar trends were detected at the postchemotherapy time point for both RFS (HR, 4.51; 95% CI, 2.27–8.94; *p* < 0.001, Fig. 2A) and OS (HR, 4.41; 95% CI, 0.52–37.29; *p* = 0.17, Fig. 2B).

### Lead time analysis

A summary of the lead time by biomarker detection preceding radiographic progression was presented in Fig. 2C. All of the 9 eligible studies referred to *ctDNA* detection, with a total of 148 patients suffering disease progression [12, 13, 15, 68, 69, 71, 72, 74, 75]. Molecular recurrence was detected by next-generation sequencing (NGS) approaches and cancer personalized profiling by deep sequencing (CAPP-Seq), circulating single-molecule amplification and resequencing technology (cSMART), and multiplex polymerase chain reaction NGS (mPCR-NGS) platforms. The reported lead time ranged from 0 to 795 days. Taken together, our study found an average lead time of 179 ± 74 days.

### Predictive value

**Fig. 3:**
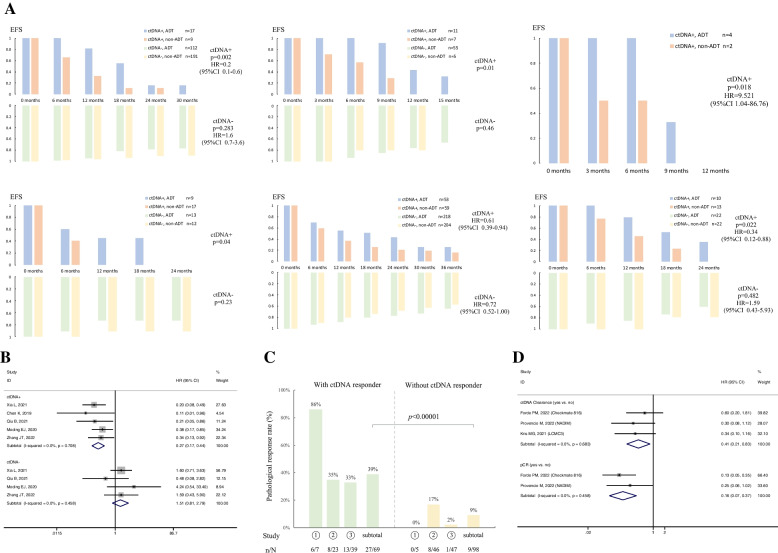
Analysis of predictive value and treatment response evaluation

The detailed data for predictive value analysis of *MRD* detection in adjuvant therapy guidance after lung cancer resection are presented in Fig. 3A [13, 66, 69, 72, 75, 76]. All of the 6 eligible cohorts explored the *ctDNA*-based *MRD* predictive value for adjuvant therapy (Additional file 4: Table S2). In this series, adjuvant therapy was found to confer a survival benefit for patients with detectable *ctDNA*-based *MRD*, while adjuvant therapy could not improve survival for undetectable *MRD* patients. Furthermore, the forest plot showed that the application of adjuvant therapy significantly benefited long-term survival in patients with *ctDNA*-based *MRD* + (RFS, HR, 0.27; 95% CI, 0.17–0.44; *p* < 0.001), while an opposite tendency was detected for *MRD − *patients (RFS, HR, 1.51; 95% CI, 0.81–2.79; *p* = 0.19) (Fig. 3B).

### Treatment response evaluation

There were 3 studies eligible for the analysis of correlation between pathological response and *ctDNA* responder for neoadjuvant therapy (Additional file 2: Table S7) [71, 77, 78]. The percentage of patients with a major pathological response (MPR) or pathologic complete response (pCR) was higher among those with *ctDNA* responder (33% ~ 86%) than among those without *ctDNA* responder (0 ~ 17%) (Fig. 3C). Further Fisher’s exact test indicated a strong correlation between a pathological response and *ctDNA* responder (*p* < 0.00001). In addition, significantly improved long-term survivals were both observed for patients with pCR (HR, 0.41, 95% CI, 0.21–0.83, *p* < 0.05) and *ctDNA* clearance (HR, 0.16, 95% CI, 0.07–0.37, *p* < 0.001) after neoadjuvant therapy (Fig. 3D) [73, 77, 78].

## Discussion

The study is a comprehensive systematic review and meta-analysis to evaluate the clinical utility of liquid biopsy focusing on the early-stage NSCLC, regarding the commonly detected biomarkers, CTCs, *ctDNA*, methylation signatures, and *microRNAs*. The present study indicated liquid biopsy could reliably facilitate more precision and effective management of early-stage NSCLC.

The early detection of lung cancer is critical for reducing its mortality and morbidity. In recent decades, many studies have investigated the screening and diagnostic values of different biomarkers, including *ctDNA*, CTCs, *DNA* methylation, and *microRNAs*. Our analysis indicated that these common biomarkers could provide similar diagnostic accuracy for early-stage NSCLC, with acceptable effectiveness in AUCs ranging from 0.84 to 0.87. With the development of platforms and technology, *ctDNA* analysis has provided a practical approach to the noninvasive detection of early-stage tumors [14, 35, 39]. In addition, the analysis of *cfDNA* fragmentomics profiles was newly highlighted as the novel approach and pattern for the accurate screening, early detection, and monitoring of human cancer [79, 80]. With the advantages of an early appearance in the disease course, cancer specificity, biological stability, and ready accessibility in bodily fluids [81], aberrant *DNA* methylation analysis combined with machine learning provided the opportunity to overcome the challenge of the low abundance of *ctDNA* in plasma samples [28, 29, 34, 58, 82, 83]. CTCs in the blood could serve as effective screening and diagnostic markers to discriminate small malignant pulmonary nodules from benign lesions [54, 56]. The profiling of circulating *microRNAs* has promising accuracy for discriminating lung cancer patients from healthy controls and could be developed as a supplement in future screening [51, 84]. Recently, integrative multi-analytical models combining clinical features and multiple biomarkers have achieved a better balance of predictive sensitivity and specificity [16, 40, 53]. Multiomics analysis combining *ctDNA* detection with methylation, exosomes, circulating *microRNAs*, circulating tumor cells, metabonomics, and molecular imaging methods should be considered to improve the efficacy and provide more practical value for clinical applications.

In this study, there was a lower diagnostic accuracy for stage I NSCLC. The low concentration of *cfDNA* molecules in plasma introduces a biological limitation for detecting early-stage tumors [85, 86]. Similar to the previous studies [85, 87], our analysis pooled a median *cfDNA* concentration of 8.64 ng/ml in early-stage NSCLC and suggested that TNM stage I, T1 stage, N0 stage, adenocarcinoma, young age, and nonsmoking were associated with significantly lower *cfDNA* concentrations. The summarized data on the limited tumor burden highlighted the physical limitation of *ctDNA* analyses, particularly for the early detection of NSCLC patients with T1a-c stage. Additionally, clonal hematopoiesis of undetermined potential (*CHIP*) during aging is a common confounding factor influencing *ctDNA* detection. Deep sequencing of both white blood cell *DNA* and *cfDNA* might be required to identify and filter out *CHIP*-related mutations to reduce false-positive *ctDNA* detection rates [85].

With the development of new technologies and platforms, the feasibility and efficacy of *MRD* assessment and postoperative disease monitoring for early-stage NSCLC have gradually been investigated [85, 88, 89]. It is now widely recognized that patients with positive *ctDNA* and *MRD* detection after curative intent treatment have a worse prognosis than those with undetectable *MRD* [12, 15, 66, 72, 75]. In addition, the promising *ctDNA MRD* analysis for the early detection of disease recurrence preceding radiographic progression by an average lead time of 179 ± 74 days could reliably facilitate more effective interventions, offering the chance for earlier treatment strategy decision-making and to treat patients at their lowest tumor burden [12, 13, 15, 71, 74]. Although the other biomarkers including CTC, *DNA* methylation and *microRNAs* also showed the promising prognostic values, there is still lack of clinical cohort regarding their *MRD* detection during post-treatment monitoring [59, 60, 84].

A summary of the four different *MRD* detection patterns is presented in Table 1. For assay design, a tumor-naive panel involves sequencing several genes commonly known to be mutated in lung cancer, and thus there is no need for individualized tumor information [90]. The novel CAPP-Seq strategy has developed and achieved the enhancements in *ctDNA* analysis, with the ultrasensitive detection efficiency under the ultralow detection limit [14, 91]. Correspondingly, tumor-informed assays were designed based on the whole exome/genome sequencing of tumor tissues and tumor-adjacent normal tissues. The personalized panels take advantage of accurately tracking a larger number of mutations for each patient and improving the sensitivity of *ctDNA* detection [92]. However, the method is more expensive and has limitations in detecting de novo resistance/oncogenic alterations. The novel *MRDetect* model demonstrated that increased breadth or an expanded number of targeted mutations via genome wide mutational integration could effectively overcome the limitation of *cfDNA* abundance with a modest sequencing depth [11]. Honestly, *DNA* methylation analysis was proved to provide the promising efficacy in cancer detection and screening [82, 83], but there is still a lack of large-scale validations and clinical implementations regarding their *MRD* detection during post-treatment monitoring in early-stage lung cancer [93]. The currently ongoing prospective cohort, which was designed to investigate the feasibility of tumor-informed methylation-based *MRD* detection and postoperative cancer surveillance, is fully expected [94].

**Table 1:** Summary of *MRD* detection patterns

Pattern	Tissue	Example	Advantage	Disadvantage
Fixed panel	No	CAPP-Seq	Tumor-agnostic, stable, time-effective	Miss some personalized information
Personalized panel	Yes	TRACERx (Signatera ArcherDx)	Tumor-informed design	Tissue-relied, expensive, longer periods
WGS-based panel	Yes	*MRDetect*	Modest sequencing depth, comprehensive genetic information	Limited confidence in sensitivity to individual site
Methylation-based panel	Yes	MEDAL	Early onset, more variants and sites	Regions optimization needed

*MRD* molecular residual disease, *WGS* whole-genome sequencing, *CAPP-Seq* cancer personalized profiling by deep sequencing, *TRACERx* tracking non-small cell lung cancer evolution through therapy (Rx), *MEDAL* methylation-based dynamic analysis for lung cancer

Increasing evidence indicates that *ctDNA*-based *MRD* detection could provide good prognostic value for early-stage NSCLC after surgical resection and adjuvant therapy. However, evidence of the predictive value of *MRD* detection for adjuvant therapy guidance is still lacking. Our results indicated that the application of adjuvant therapy significantly benefited long-term survival in patients with *ctDNA*-based *MRD* + , while adjuvant therapy could not improve survival for *MRD − *patients [13, 66, 69, 72, 75, 76]. These results clearly revealed that longitudinal *MRD* monitoring could provide clinical utility for individualized patient care with adjuvant therapies and could efficiently avoid overtreatment of low-risk patients. However, the evidence should be interpreted cautiously because of the small sample sizes and lack of prospective interventional study designs. Thus, randomized controlled trials are necessary to confirm the predictive value of *MRD*. The MERMAID-1 trial [95] is ongoing to assess the efficacy of adjuvant durvalumab combined with chemotherapy in postsurgical *MRD* + patients with stage II–III NSCLC. Meanwhile, the ongoing MERMAID-2 trial [96] is designed to evaluate the efficacy and safety of durvalumab adjuvant therapy in stage II–III NSCLC patients who become *MRD* + during the surveillance period after curative intent therapy. The results of these relevant trials are highly anticipated, and high-level evidence is urgently awaited (Table 2) [97–99].

**Table 2:** Summary of the ongoing trails

ID	Identifier	Design	Status	Participants	Sample	Stage	Intervention	Outcome measures
NCT04385368 [95]	MERMAID-1	RCT	Recruiting	NSCLC with *MRD* + after surgery	332	II-III	Drug: adjuvant durvalumab + chemotherapy Other: placebo + adjuvant chemotherapy	DFS; OS
NCT04642469 [96]	MERMAID-2	RCT	Recruiting	NSCLC with *MRD* + after surgery	284	II-III	Drug: durvalumab Other: placebo	DFS; OS
NCT04367311 [97]	BTCRC-LUN19-396	Non-RCT	Recruiting	NSCLC after surgery	100	I-IIIA	Adjuvant chemotherapy + atezolizumab	Percentage of patients with undetectable *ctDNA*; DFS
NCT04585477 [98]	LUN0115	Non-RCT	Recruiting	NSCLC after surgery or SBRT	80	I-III	*MRD* + cohort 1: adjuvant durvalumab *MRD − *cohort 2: SoC	Decrease in *ctDNA* level; OS; DFS; irAEs
NCT04585490 [99]	LUN0114	Non-RCT	Recruiting	Unresectable NSCLC	48	III	*MRD* + cohort 1: chemotherapy + durvalumab *MRD − *cohort 2: durvalumab	Change in *ctDNA* Level; PFS; OS; irAEs

*RCT* randomized controlled trial, *NSCLC* non-small cell lung cancer, *MRD* minimal residual disease, *SoC* standard of care, *ctDNA* circulating tumor *DNA*, *OS* overall survival, *DFS* disease-free survival, *PFS* progression-free survival, *SBRT* stereotactic body radiation therapy, *irAEs* immune-related adverse events

Recently, the clinical utility of *ctDNA* in predicting the response to neoadjuvant treatments and assessing the prognosis of NSCLC has gradually been explored [71, 77, 100]. The NADIM trial [73] reported for the first time a significant association between *ctDNA* levels after neoadjuvant chemoimmunotherapy and survival outcomes in operable NSCLC. The data were supported by 3-year OS and revealed that *ctDNA* outperformed radiologic assessments in the prediction of survival, which highlighted the usefulness of *ctDNA* as an early surrogate end point for neoadjuvant treatment. Likewise, CheckMate 816 [78] showed that *ctDNA* clearance was associated with a pathologic response, and event-free survival appeared longer in patients with *ctDNA* clearance than in those without, suggesting that clearance during neoadjuvant therapy may be an early predictor of favorable outcomes. Correspondingly, our study detected a strong correlation between pathological response and *ctDNA* clearance, and significantly improved long-term survivals were both observed for patients with pCR and *ctDNA* clearance after neoadjuvant therapy. These results revealed the effective utility of *ctDNA* status and dynamics analysis and provided a landmark approach for evaluating the response of therapeutic outcomes for resectable NSCLC patients treated with neoadjuvant therapy. We require more evidence and data regarding the treatment response evaluation of *ctDNA* analysis in lung cancer disease monitoring.

## Limitations

Several limitations of this study should be considered. First, the types of liquid biomarkers, detection technologies, and platforms and the lack of a standardized *ctDNA* detection manual all contribute to the great heterogeneity among the included studies. Second, there were 10 extensive stage patients enrolled in our analysis [16, 63, 69], but their impact may be negligible. In addition, even though we have completed a comprehensive and systematic search of the literature, publication bias is still inevitable. Finally, some studies did not report detailed information on histology and radiology, and further analysis was therefore limited.

## Conclusions

In conclusion, our study indicated liquid biopsy could reliably facilitate more precision and effective management of early-stage NSCLC, regarding the commonly detected biomarkers, CTCs, *ctDNA*, methylation signatures, and *microRNAs*. The lead time analysis suggested *ctDNA* detection monitoring could provide the opportunity for earlier interventions during disease surveillance. Improvement of liquid biopsy techniques and detection approaches and platforms are still needed, and higher-quality trails are required to provide more rigorous evidence prior to their routine clinical application.

## Supplementary Information

**Additional file 1: Table S1.** PRISMA 2020 checklist.

**Additional file 2: Table S1.** Example of search strategy as used for the PubMed database. **Table S2.** Description of included studies. **Table S3.** Risk of bias assessment of included studies using QUADAS-2 tool. **Table S4.** Assessment of bias by Newcastle-Ottawa scale. **Table S5.** Diagnostic performance of different biomarkers in early-stage NSCLC. **Table S6.** Analysis of concentration of cell-free *DNA*. **Table S7.** Concordance between *ctDNA* and pathological response to neoadjuvant therapy.

**Additional file 3: Figure S1.** PRISMA flow diagram. **Figure S2.** Deeks’ funnel plot in diagnostic analysis. **Figure S3.** Funnel plot in prognostic analysis of RFS at (A) preoperative and (B) postoperative time point; and OS at (C) preoperative and (D) postoperative time point.

**Additional file 4: Table S1.** Summary of concentration of *cfDNA* in eligible cohorts. **Table S2.** Predictive value analysis.

## Data Availability

All data generated or analyzed during this study are included in this published article and its supplementary information files.

## Abbreviations

AUC: Area under the curve
CAPP-seq: Cancer personalized profiling by deep sequencing
*cfDNA*: Circulating cell-free *DNA*
*CHIP*: Clonal hematopoiesis of undetermined potential
CI: Confidence interval
cSMART: Circulating single-molecule amplification and resequencing technology
CTC: Circulating tumor cell
*ctDNA*: Circulating tumor *DNA*
HR: Hazard ratio
LDCT: Low-dose computed tomography
LUAD: Lung adenocarcinoma
LUSC: Lung squamous cell carcinoma
MEDAL: Methylation based dynamic analysis for lung cancer
MIDAS: Stata module for meta-analytical integration of diagnostic test accuracy studies
mPCR-NGS: Multiplex PCR NGS platforms
MPR: Major pathological response
*MRD*: Molecular residual disease
NGS: Next-generation sequencing
NSCLC: Non-small cell lung cancer
OS: Overall survival
PCR: Polymerase chain reaction
pCR: Pathologic complete response
RFS: Recurrence-free survival
SROC: Summary receiver operative curve
TNM: Tumor, node, metastasis classification
TRACERx: Tracking non-small cell lung cancer evolution through therapy (Rx) study
WGS: Whole-genome sequencing
