# Supplementary material for: Potential clinical utility of liquid biopsy in early-stage non-small cell lung cancer
Source: BMC Med. 2022 Dec 14;20:480. doi: 10.1186/s12916-022-02681-x (PMC9749360; doi:10.1186/s12916-022-02681-x)
Supplement: Supplementary file 2 — Additional file 2: Table S1. Example of search strategy as used for the PubMed database. Table S2. Description of included studies. Table S3. Risk of bias assessment of included studies using QUADAS-2 tool. Table S4. Assessment of bias by Newcastle-Ottawa scale. Table S5. Diagnostic performance of different biomarkers in early-stage NSCLC. Table S6. Analysis of concentration of cell-free DNA. Table S7. Concordance between ctDNA and pathological response to neoadjuvant therapy. [file 12916_2022_2681_MOESM2_ESM.docx]

**Additional file 2: Tables S1-S7. Table S1-**Example of search strategy as used for the PubMed database. **Table S2-**Description of included studies. **Table S3-**Risk of bias assessment of included studies using QUADAS-2 tool. **Table S4-**Assessment of bias by Newcastle-Ottawa scale. **Table S5-**Diagnostic performance of different biomarkers in early-stage NSCLC. **Table S6-**Analysis of concentration of cell-free *DNA*. **Table S7-**Concordance between *ctDNA* and pathological response to neoadjuvant therapy.

**Table S1.** Example of search strategy as used for the PubMed database

| **Search strategy (PubMed database)** |
| --- |
| ***Lung Neoplasm***  **#1** (("Lung Neoplasms"[Mesh]) OR “Lung Neoplasm” OR “Pulmonary Neoplasm” OR “Lung Cancer” OR “Pulmonary Cancer” OR “Lung Tumor” OR “Lung Carcinoma” OR “Pulmonary Carcinoma” OR “Lung Disease” OR “Cancer of Lung” OR “Pulmonary Malignancy” OR “Pulmonary Tumor”) **[Title/Abstract]**  ***Liquid Biopsy***  **#2** (("Liquid Biopsy"[Mesh]) OR “Liquid Biopsy” OR “Biopsy, Liquid” OR “Liquid Biopsies” OR “Biopsies, Liquid” OR “Fluid Biopsy”) **[Title/Abstract]**  **#3** (("Circulating Tumor *DNA*"[Mesh]) OR “Circulating Tumor *DNA*” OR “*DNA*, Circulating Tumor” OR “Tumor *DNA*, Circulating” OR “Cell-Free Tumor *DNA*” OR “Cell Free Tumor *DNA*” OR “*DNA*, Cell-Free Tumor” OR “Tumor *DNA*, Cell-Free” OR “*ctDNA*”) **[Title/Abstract]**  **#4** ((“Cell Free Nucleic Acids” [Mesh]) OR “Cell Free Nucleic Acids” OR “Circulating Cell Free Nucleic Acid” OR “Circulating Nucleic Acids” OR “Cell Free Nucleic Acid” OR “Circulating Cell Free Nucleic Acids” OR “Circulating Nucleic Acid” OR “Cell Free *DNA*” OR “*cfDNA*” OR “*cirDNA*” OR “Cell Free Deoxyribonucleic Acid” OR “Circulating *DNA*” OR “Cell Free *RNA*” OR “*cfRNA*” OR “*cirRNA*” OR “*ctRNA*” OR “Cell Free Ribonucleic Acid” OR “Circulating *RNA*”) **[Title/Abstract]**  **#5** ((“Neoplastic cells, Circulating” [Mesh]) OR “Neoplasm Circulating Cell” OR “Circulating Neoplastic Cell” OR “Circulating Tumor Cell” OR “Embolic Tumor Cell” OR “Tumor Embolism”) **[Title/Abstract]**  **#6** ((“*DNA* Methylation” [Mesh]) OR “*DNA* Methylation”) **[Title/Abstract]**  **#7** (**#2** OR **#3** OR **#4** OR **#5** OR **#6**)  ***Final search strategies:***  **#1** AND **#7** |

| **Table S2.** Description of included studies | | | | | | | | | | | | | | | | | | |
| --- | --- | --- | --- | --- | --- | --- | --- | --- | --- | --- | --- | --- | --- | --- | --- | --- | --- | --- |
| Author [Ref.] | Year | Period | Country | Study Type | Sample | Age | Male, n (%) | Smoker, n (%) | Tumor Stage | TNM Edition | Histology | Blood Type | Biomarker | Method | TP | FP | FN | TN |
|  |  |  |  |  |  | Patient/Control | Patient/Control | Patient/Control | I/II/III |  | Ad/Sq/Others |  |  |  |  |  |  |  |
| **Diagnosis values** | | | | | | | | | | | | | | | | |  |  |
| Sozzi G [27] | 1999 | NA | Italy | NA | 98 | 63/51 | 55(65.5)/10(71.4) | NA/10(71.4) | 40/25/19 | - | 32/45/11 | Plasma | *ctDNA* | PCR | 33 | 0 | 51 | 14 |
| Ostrow KL [28] | 2010 | NA | USA | Prospective | 139 | 69/53 | 37(62.7)/39(48.8) | NA/78(97.5) | 47/2/10 | 7 | 43/6/10 | Plasma | Methylation | PCR | 42 | 23 | 17 | 57 |
| Begum S [29] | 2011 | NA | USA | NA | 88 | 65/NA | 40(69.0)/NA | NA | 41/17/- | 7 | 36/26/14 | Serum | Methylation | PCR | 43 | 7 | 15 | 23 |
| Kneip C [30] | 2011 | NA | Germany | NA | 264 | NA | NA | NA | 34/27/48 | 7 | 20/31/58 | Plasma | Methylation | PCR | 53 | 16 | 56 | 139 |
| Wozniak MB [31] | 2015 | 2006-2012 | Russia | Retrospective | 200 | 62.6/60.1 | 86(86.0)/71(71.0) | 89(89)/60(60.0) | 49/21/30 | 7 | 35/65/- | Plasma | *MicroRNA* | PCR | 83 | 16 | 17 | 84 |
| Halvorsen AR [32] | 2016 | 2006-2011 | Norway | Prospective | 137 | 62.6/57.6 | 72(91.1)/34(58.6) | NA/58(100.0) | 65/14/- | 7 | 98/2/- | Serum | *MicroRNA* | PCR | 67 | 15 | 12 | 43 |
| Balgkouranidou I [33] | 2016 | NA | Greece | Retrospective | 92 | NA | NA | NA | 14/29/- | 7 | 22/21/- | Plasma | Methylation | PCR | 27 | 1 | 16 | 48 |
| Ooki A [34] | 2017 | NA | USA | Retrospective | 125 | 70.9/66.7 | 37(44.6)/15(35.7) | 66(79.5)/30(71.4) | 83/-/- | 7 | 43/40/- | Serum | Methylation | PCR | 55 | 12 | 28 | 30 |
| Phallen J [35] | 2017 | NA | USA | Retrospective | 109 | 58/44 | 46(70.8)/0(0.0) | NA | 29/32/4 | 7 | 36/14/15 | Plasma | *ctDNA* | TEC-Seq NGS | 39 | 0 | 26 | 44 |
| Powrózek T [36] | 2017 | 2015-2016 | Poland | NA | 156 | 62/60 | 42(75.0)/63(63.0) | NA | 14/23/19 | 7 | 26/30/- | Plasma | *MicroRNA* | PCR | 42 | 18 | 14 | 82 |
| Sun Y [37] | 2018 | 2014.01-2016.12 | China | Retrospective | 56 | 69.5/58.3 | 17(60.7)/13(46.4) | NA | 15/7/6 | 7 | 28/-/- | Plasma | *MicroRNA* | PCR | 26 | 2 | 2 | 26 |
| Wei F [38] | 2018 | 2014.12-2016.03 | USA | Prospective | 44 | NA | 9(42.9)/12(52.2) | NA | 19/3/- | 8 | 21/-/- | Plasma | *ctDNA* | eLB | 18 | 2 | 3 | 21 |
| Wan Y [39] | 2018 | 2016.12-2018.02 | China | Retrospective | 353 | 57/61 | 119(41.9)/26(37.7) | 92(32.4)/20(29.0) | 107/117/- | 8 | 231/35/18 | Plasma | *ctDNA* | ARMS-PCR | 73 | 2 | 211 | 67 |
| Cohen JD [40] | 2018 | NA | USA | Retrospective | 916 | 69/55 | 63(60.6)/434(53.4) | NA | 46/27/31 | 7 | NA | Plasma | Multi-analyte | PCR | 61 | 7 | 43 | 805 |
| Liang W [41] | 2019 | NA | China | Retrospective | 54 | NA | NA | NA | 27/-/- | 8 | NA | Plasma | Methylation | NGS | 21 | 4 | 6 | 23 |
| Peng M [42] | 2019 | NA | China | Prospective | 189 | 59.1/50.1 | 82(61.7)/31(55.4) | 48(36.1)/18(32.1) | 87/29/17 | 8 | 100/28/5 | Plasma | *ctDNA* | PCR | 95 | 2 | 38 | 54 |
| Villalba M [43] | 2019 | NA | Spain | Retrospective | 41 | NA | NA | NA | 8/8/- | 8 | NA | Plasma | Methylation | PCR | 14 | 9 | 2 | 16 |
| Liu J [44] | 2019 | 2015.01-2018.03 | China | Prospective | 96 | NA | NA/29(40.8) | NA | 25/-/- | 8 | NA | Blood | CTC | PCR | 17 | 7 | 8 | 64 |
| Yang Z [45] | 2019 | 2015.07-2017.07 | China | NA | 50 | 51/54 | 24(61.5)/7(63.6) | 29(74.4)/8(72.7) | 39/-/- | 7 | NA | Plasma | Methylation | PCR | 28 | 1 | 11 | 10 |
| Yang Y [46] | 2019 | NA | China | Retrospective | 48 | NA | NA | NA | 24/8/- | 7 | 18/11/3 | Plasma | *ctDNA* | NGS | 19 | 5 | 13 | 11 |
| Ghany SMA [47] | 2020 | 2015.02-2017.01 | Egypt | Retrospective | 104 | 63.2/55.6 | 49(70.0)/17(50.0) | 59(84.3)/17(50.0) | -/28/42 | 8 | 24/35/11 | Plasma | *MicroRNA* | PCR | 56 | 14 | 14 | 20 |
| Zhang ZJ [48] | 2020 | 2019.04-2019.07 | China | Prospective | 386 | NA | NA | NA | 104/-/- | 8 | NA | Serum | *MicroRNA* | PCR | 73 | 51 | 31 | 231 |
| He Y [49] | 2020 | 2014.01-2017.03 | China | Retrospective | 96 | 61.4/NA | 45(45.8)/42(58.3) | 11(45.8)/NA | 18/6/- | 8 | 18/6/- | Blood | CTC | ICC | 15 | 0 | 9 | 72 |
| Liu WR [50] | 2020 | 2018.05-2018.12 | China | Prospective | 310 | 56/45 | 90(38.8)/43(55.1) | 74(31.9)/21(26.9) | 232/-/- | 8 | 222/-/10 | Blood | CTC | IF | 156 | 15 | 76 | 63 |
| Wang W [51] | 2020 | 2016.07-2018.03 | China | Retrospective | 40 | NA | 16(61.5)/7(50.0) | 9(34.6)/5(35.7) | 21/5/- | 8 | 22/3/1 | Plasma | *MicroRNA* | PCR | 13 | 1 | 13 | 13 |
| Chabon JJ [14] | 2020 | 2009.11-2018.12 | USA | Prospective | 160 | 70/69 | 63(60.6)/35(62.5) | 83(79.8)/56(100.0) | 49/28/27 | 7 | 71/23/10 | Plasma | *ctDNA* | PCR | 53 | 1 | 51 | 55 |
| Chen C [52] | 2020 | 2016.12-2018.04 | China | NA | 246 | 58.8/52.5 | 84(51.5)/52(62.7) | NA | 163/-/- | 8 | 139/22/2 | Plasma | Methylation | PCR | 147 | 24 | 16 | 59 |
| Liu QX [53] | 2021 | 2019.02-2019.12 | China | Prospective | 29 | 59.1/52.5 | 3(20.0)/7(50.0) | 2(13.3)/3(21.4) | 15/-/- | 8 | 15/-/- | Plasma | Multi-analyte | NGS | 12 | 2 | 3 | 12 |
| Ye M [54] | 2021 | 2019.01-2020.01 | China | Prospective | 125 | 50.9/53.7 | 23(28.4)/24(54.5) | 14(17.3)/17(38.6) | 81/-/- | 8 | NA | Blood | CTC | IF | 57 | 6 | 24 | 38 |
| Feng M [55] | 2021 | 2019.10-2020.01 | China | Prospective | 205 | 62 | 97(47.3) | 51(30.4)/(30.3) | 168/-/- | 8 | 156/8/4 | Blood | CTC | IF | 145 | 8 | 23 | 29 |
| Liu C [56] | 2021 | 2019.05-2019.10 | China | Prospective | 53 | 61/59 | 20(48.8)/9(75.0) | NA | 34/6/1 | 8 | 38/3/- | Blood | CTC | IF | 29 | 3 | 12 | 9 |
| Liang W [57] | 2021 | 2017.05-2019.02 | China | Retrospective | 220 | 55.8 | 81(50.6)/33(55.0) | 49(30.6)/18(30.0) | 138/6/2/14^a^ | 8 | 139/10/11 | Plasma | Methylation | PCR | 155 | 35 | 5 | 25 |
| Liang N [58] | 2021 | NA | China | Retrospective | 569 | 56.5/54.0 | 153(49.7)/144(55.2) | 137(44.5)/105(40.2) | 199/66/43 | 8 | 235/56/17 | Plasma | Methylation | NGS | 194 | 10 | 114 | 251 |
| Chen K [16] | 2021 | 2013.12-2018.12 | China | Retrospective | 134 | 64.0/54.5 | 45(60.8)/30(50.0) | 33(44.6)/15(25.0) | 48/13/7/6^b^ | 8 | 53/17/4 | Plasma | Multi-analyte | NGS | 57 | 25 | 17 | 35 |
| **Prognosis values** |  |  |  |  |  |  |  |  |  |  |  |  |  |  | Follow-up (m) | |  |  |
| Hu [59] | 2010 | 2003-2007 | China | Prospective | 303 | NA | NA | NA | NA | 7 | NA | Serum | *MicroRNA* | PCR | 49.5 | |  |  |
| Balgkouranidou I [33] | 2016 | NA | Greece | Retrospective | 44 | NA | 34(77.3) | NA | 14/30/- | 7 | 22/21/1 | Plasma | Methylation | PCR | 45(1-73) | |  |  |
| Chaudhuri AA [15] | 2017 | 2010.07-2016.03 | USA | Retrospective | 40 | 66.5 | 27(67.5) | 35(87.5) | 7/7/26 | 7 | 19/15/6 | Plasma | *ctDNA* | CAPP-Seq | 35.1(6.9-56) | |  |  |
| Dandachi N [60] | 2017 | 2015.04-2016.05 | Austria | Prospective | 40 | 67 | 16(40.0) | 13(32.5) | 19/9/12 | 7 | 40/-/- | Blood | CTC | IF | 16.03 | |  |  |
| Abbosh C [12] | 2017 | NA | UK | Prospective | 24 | 67.5 | 16(66.7) | 23(95.8) | 10/10/4 | 8 | 16/8/- | Plasma | *ctDNA* | mPCR-NGS | 25.4 | |  |  |
| Chen K [13] | 2019 | 2016.12-2017.09 | China | Prospective | 26 | 65 | 18(69.2) | 15(57.7) | 4/5/17 | 8 | 12/12/2 | Plasma | *ctDNA* | cSMART | 17.7(16.0-22.7) | |  |  |
| de Miguel-Pérez D [61] | 2019 | 2012.12-2015.02 | Spain | Prospective | 97 | 66.1 | 84(86.6) | 88(90.7) | 44/25/18 | 8 | 47/50/- | Blood | CTC | IF | 28 | |  |  |
| Isaksson S [62] | 2019 | 2005-2014 | Sweden | Retrospective | 58 | 69 | 20(34.5) | 49(84.5) | 34/14/10 | 8 | 58/-/- | Plasma | *ctDNA* | PCR | 39.6(8.4-82.8) | |  |  |
| Peng M [63] | 2020 | 2014.02-2015.12 | China | Prospective | 77 | 60.3 | 56(72.7) | 28(36.4) | 40/18/17/2^b^ | 8 | 40/30/7 | Plasma | *ctDNA* | cSMART | 46 | |  |  |
| Chabon JJ [14] | 2020 | 2009.11-2018.12 | USA | Prospective | 85 | 70 | 50(58.8) | NA | 48/21/16 | 8 | 63/18/4 | Plasma | *ctDNA* | PCR | 30.1 | |  |  |
| Yang W [64] | 2020 | 2016.07-2019.03 | China | Prospective | 82 | 55.8 | 33(40.2) | 23(28.0) | 82/-/- | 8 | 82/-/- | Plasma | *ctDNA* | NGS | 22.8(8.0-45.1) | |  |  |
| Kuang PP [65] | 2021 | 2016.07-2019.02 | China | Prospective | 38 | 57.5 | 22(57.9) | 17(44.7) | 7/16/15 | 8 | 23/6/9 | Plasma | *ctDNA* | NGS | 15.8(3.7-36.7) | |  |  |
| Xia L [66] | 2021 | 2017.09-2020.05 | China | Prospective | 330 | 59 | 161(48.8) | 112(33.9) | 221/60/49 | 8 | 280/43/7 | Plasma | *ctDNA* | NGS | 35.6(11.4-44.7) | |  |  |
| Ito M [67] | 2021 | 2010.04-2018.12 | Japan | Retrospective | 100 | 70.8 | 40(40.0) | 31(31.0) | 98/2/0 | 8 | 100/-/- | Serum | *ctDNA* | PCR | NA (at least 60) | |  |  |
| Li N [68] | 2021 | 2016.06-2019.02 | China | Prospective | 119 | 57 | 70(58.8) | 54(45.4) | 77/24/18 | 7 | 87/21/11 | Plasma | *ctDNA* | NGS | 30.7(28.8-32.6) | |  |  |
| Qiu B [69] | 2021 | 2018-2020 | China | Prospective | 89 | 64 | 67(75.3) | 61(68.5) | 12/41/48/2^b^ | 8 | 60/38/5 | Plasma | *ctDNA* | NGS | 12.6(1.1-19.7) | |  |  |
| Guo K [70] | 2021 | 2014.02-2015.06 | China | Prospective | 174 | NA | 73(42.0) | 43(24.7) | 7//20 | 7 | 161//13 | Plasma | *ctDNA* | PCR | NA (at least 60) | |  |  |
| Gale D [74] | 2022 | 2015.01-2020.02 | UK | Prospective | 88 | 72.5 | 45(51.1) | 79(89.8) | 43/25/20 | 7 | 55/27/6 | Plasma | *ctDNA* | NGS | 36(1.4-60) | |  |  |
| Yue D [71] | 2022 | 2018.08-2019.07 | China | Retrospective | 22 | 61.4 | 17(77.27) | 18(81.82) | 5/4/13 | 8 | -/14/- | Plasma | *ctDNA* | NGS | 17.7(2.8-23.4) | |  |  |
| Zhang JT [72] | 2022 | 2019.03-2021.01 | China | Prospective | 261 | 62 | 158(60.5) | 97(37.2) | 163/53/45 | 8 | 203/33/25 | Plasma | *ctDNA* | NGS | 19.7 | |  |  |
| Provencio M [73] | 2022 | 2017.04-2018.08 | Spain | Prospective | 46 | 63 | 34(74.0) | 46(100) | -/-/46 | 7 | 26/16/4 | Plasma | *ctDNA* | NGS | 24.0 | |  |  |
| Age or Follow-up (month) depicted in mean, median or median (range).  Ad, adenocarcinoma; Sq, squamous cell carcinoma; TP, true positive; FP, false positive; FN, false negative; TN, true negative; PCR, polymerase chain reaction; NGS, next-generation sequencing; TEC-Seq, targeted error correction sequencing; eLB, electric field-induced release and measurement liquid biopsy; ARMS-PCR, amplification-refractory mutation system-based PCR; ICC, Immunocytochemistry; IF, Immunofluorescence; CAPP-Seq, CAncer personalized profiling by deep sequencing; mPCR-NGS, multiplex-PCR NGS; cSMART, circulating single-molecule amplification and resequencing technology; NA, not available.  ^a^ Patients with unknown stage.  ^b^ Patients with stage IV. | | | | | | | | | | | | | | | | | | |

| **Table S3.** Risk of bias assessment of included studies using QUADAS-2 tool | | | | | | | | |
| --- | --- | --- | --- | --- | --- | --- | --- | --- |
| **Study** | | **Risk of bias** | | | | **Applicability concerns** | | |
| YEAR | AUTHOR | PATIENT SELECTION | INDEX TEST | REFERENCE STANDARD | FLOW AND TIMING | PATIENT SELECTION | INDEX TEST | REFERENCE STANDARD |
| 1999 | Sozzi G [27] | N | N | Y | U | N | U | Y |
| 2010 | Ostrow KL [28] | U | Y | Y | Y | U | Y | Y |
| 2011 | Begum S [29] | U | Y | Y | Y | U | Y | Y |
| 2011 | Kneip C [30] | U | Y | Y | U | U | Y | Y |
| 2015 | Wozniak MB [31] | N | Y | Y | U | U | Y | Y |
| 2016 | Halvorsen AR [32] | Y | Y | Y | U | Y | Y | Y |
| 2016 | Balgkouranidou I [33] | N | Y | Y | Y | N | Y | Y |
| 2017 | Ooki A [34] | U | Y | Y | U | U | Y | Y |
| 2017 | Phallen J [35] | U | Y | Y | U | U | Y | Y |
| 2017 | Powrózek T [36] | Y | Y | Y | U | Y | Y | Y |
| 2018 | Sun Y [37] | Y | Y | Y | Y | U | Y | Y |
| 2018 | Wei F [38] | Y | Y | Y | U | U | Y | Y |
| 2018 | Wan Y [39] | Y | U | Y | N | U | U | Y |
| 2018 | Cohen JD [40] | Y | Y | Y | U | Y | Y | Y |
| 2019 | Liang W [41] | U | U | Y | U | Y | Y | Y |
| 2019 | Peng M [42] | Y | Y | Y | Y | Y | Y | Y |
| 2019 | Villalba M [43] | N | U | Y | N | Y | Y | Y |
| 2019 | Liu J [44] | Y | U | Y | U | Y | Y | Y |
| 2019 | Yang Z [45] | Y | U | Y | U | Y | Y | Y |
| 2019 | Yang Y [46] | U | U | Y | U | Y | Y | Y |
| 2020 | Ghany SMA [47] | Y | Y | Y | U | U | Y | Y |
| 2020 | Zhang ZJ [48] | U | U | Y | U | Y | Y | Y |
| 2020 | He Y [49] | Y | U | Y | U | Y | U | Y |
| 2020 | Liu WR [50] | Y | Y | Y | Y | Y | Y | Y |
| 2020 | Wang W [51] | Y | Y | Y | U | Y | Y | Y |
| 2020 | Chabon JJ [14] | Y | Y | Y | Y | Y | Y | Y |
| 2020 | Chen C [52] | Y | Y | Y | U | Y | Y | Y |
| 2021 | Liu QX [53] | Y | U | Y | Y | Y | Y | Y |
| 2021 | Ye M [54] | Y | Y | Y | Y | Y | Y | Y |
| 2021 | Feng M [55] | Y | U | Y | Y | Y | Y | Y |
| 2021 | Liu C [56] | Y | U | Y | Y | Y | Y | Y |
| 2021 | Liang W [57] | Y | Y | Y | Y | Y | Y | Y |
| 2021 | Liang N [58] | N | Y | Y | Y | Y | Y | Y |
| 2021 | Chen K [16] | Y | Y | Y | U | Y | Y | Y |
| Y, yes; N, no; U, unclear. | | | | | | | | |

| **Table S4.** Assessment of bias by Newcastle-Ottawa scale | | | | | | | | | |
| --- | --- | --- | --- | --- | --- | --- | --- | --- | --- |
| Source | Selection | | | | Comparability | Outcome | | | Score |
|  | A | B | C | D | E | F | G | H |  |
| Hu et al, [59] 2010 | * | * | * | * | * | * | * | * | 8 |
| Balgkouranidou et al, [33] 2016 | * | * | * | * | * | * | * | * | 8 |
| Chaudhuri et al, [15] 2017 | ***** | ***** | ***** | **-** | ***** | ***** | ***** | ***** | 7 |
| Dandachi et al, [54] 2017 | ***** | ***** | ***** | ***** | ***** | ***** | **-** | ***** | 7 |
| Abbosh et al, [12] 2017 | ***** | ***** | ***** | **-** | ***** | ***** | ***** | ***** | 7 |
| Chen et al, [13] 2019 | ***** | ***** | ***** | ***** | ***** | ***** | **-** | ***** | 7 |
| de Miguel-Pérez et al, [61] 2019 | ***** | ***** | ***** | ***** | ***** | ***** | ***** | ***** | 8 |
| Isaksson et al, [62] 2019 | ***** | ***** | ***** | ***** | ***** | ***** | ***** | ***** | 8 |
| Peng et al, [63] 2020 | ***** | ***** | ***** | ***** | ****** | ***** | ***** | ***** | 9 |
| Chabon et al, [14] 2020 | ***** | ***** | ***** | ***** | ****** | ***** | ***** | ***** | 9 |
| Yang et al, [64] 2020 | ***** | ***** | ***** | ***** | ***** | ***** | ***** | ***** | 8 |
| Kuang et al, [65] 2021 | ***** | ***** | ***** | ***** | ***** | ***** | **-** | ***** | 7 |
| Xia et al, [66] 2021 | ***** | ***** | ***** | ***** | ***** | ***** | ***** | ***** | 8 |
| Ito et al, [67] 2021 | ***** | ***** | ***** | ***** | ****** | ***** | ***** | ***** | 9 |
| Li et al, [68] 2021 | ***** | ***** | ***** | ***** | ****** | ***** | ***** | ***** | 9 |
| Qiu et al, [69] 2021 | ***** | ***** | ***** | ***** | ***** | ***** | **-** | ***** | 7 |
| Guo et al, [70] 2021 | ***** | ***** | ***** | ***** | ***** | ***** | ***** | ***** | 8 |
| Gale et al, [74] 2022 | ***** | ***** | ***** | ***** | ***** | ***** | ***** | ***** | 8 |
| Yue et al, [71] 2022 | ***** | ***** | ***** | ***** | ***** | ***** | **-** | ***** | 7 |
| Zhang et al, [72] 2022 | ***** | ***** | ***** | ***** | ***** | ***** | ***** | ***** | 8 |
| Provencio et al, [73] 2022 | ***** | ***** | ***** | ***** | ***** | ***** | ***** | ***** | 8 |
| A: representativeness of the exposed cohort; B: selection of the non-exposed cohort; C: ascertainment of exposure; D: demonstration that outcome of interest was not present at start of study; E: comparability of cohorts on the basis of the design or analysis (the most important factor was stage); F: assessment of outcome; G: was follow-up long enough for outcomes to occur (2-year); H: adequacy of follow up of cohorts. | | | | | | | | | |

| **Table S5.** Diagnostic performance of different biomarkers in early-stage NSCLC | | | | | | | | | | | | | | | | | | | | | | | | | | |
| --- | --- | --- | --- | --- | --- | --- | --- | --- | --- | --- | --- | --- | --- | --- | --- | --- | --- | --- | --- | --- | --- | --- | --- | --- | --- | --- |
| Biomarker | Study | Sample | | I2  SES | | P(Q-test) | | I2  SPE | | P(Q-test) | | Sensitivity | | Specificity | | PLR | | NLR | | DOR | | AUC | | P funnel plot | |  |
| **Stage I-III** | | | | | | | | | | | | | | | | | | | | | | | | | |  |
| CTC | 6 | 885 | | 78.03 | | <0.01 | | 74.14 | | <0.01 | | 0.72 [0.63,0.80] | | 0.89 [0.77,0.95] | | 6.3 [3.2,12.6] | | 0.32 [0.24,0.42] | | 20 [9,42] | | 0.84[0.81, 0.87] | | 0.44 | |  |
| *ctDNA* | 7 | 1001 | | 94.99 | | <0.01 | | 86.25 | | <0.01 | | 0.56 [0.41,0.70] | | 0.97 [0.89,0.99] | | 16.2 [ 5.3,49.9] | | 0.46 [0.33,0.64] | | 35 [11,117] | | 0.87[0.84, 0.90] | | 0.89 | |  |
| Methylation | 11 | 1888 | | 91.94 | | <0.01 | | 93.18 | | <0.01 | | 0.77 [0.66,0.85] | | 0.82 [0.70,0.90] | | 4.2 [2.7,6.6] | | 0.28 [0.20,0.40] | | 15 [9,24] | | 0.86[0.83, 0.89] | | 0.30 | |  |
| *microRNA* | 7 | 1079 | | 74.07 | | <0.01 | | 65.73 | | 0.01 | | 0.78 [0.70,0.84] | | 0.81 [0.74,0.86] | | 4.1 [3.0,5.5] | | 0.27 [0.20,0.38] | | 15 [9,25] | | 0.86[0.83, 0.89] | | 0.48 | |  |
| Multi-analyte | 3 | 1079 | | NA | | NA | | NA | | NA | | NA | | NA | | NA | | NA | | NA | | NA | | NA | |  |
| *DNA*-based | 21 | 3968 | | 94.80 | | <0.01 | | 95.03 | | <0.01 | | 0.70 [0.61,0.78] | | 0.90 [0.82,0.95] | | 7.1 [4.1,12.3] | | 0.33 [0.26,0.43] | | 21[13,36] | | 0.86[0.83, 0.89] | | 0.20 | |  |
| **Stage I** | | | | | | | | | | | | | | | | | | | | | | | | | |  |
| CTC | 4 | 584 | | 0.00 | | 0.94 | | 15.40 | | 0.31 | | 0.68 [0.63,0.73] | | 0.85 [0.79,0.89] | | 4.5 [3.2,6.3] | | 0.37 [0.32,0.44] | | 12 [8,19] | | 0.82 [0.79 - 0.85] | | 0.68 | |  |
| *ctDNA* | 5 | 416 | | 53.64 | | 0.07 | | 87.12 | | <0.01 | | 0.50 [0.40,0.59] | | 0.98 [0.84,1.00] | | 22.4 [2.9,171.5] | | 0.51 [0.43,0.62] | | 44 [6,341] | | 0.64 [0.59 - 0.68] | | 0.19 | |  |
| Methylation | 10 | 1515 | | 92.32 | | <0.01 | | 93.92 | | <0.01 | | 0.72 [0.57,0.83] | | 0.82 [0.69,0.91] | | 4.1 [2.5,6.6] | | 0.34 [0.23,0.50] | | 12 [7,20] | | 0.84 [0.80 - 0.87] | | 0.27 | |  |
| *microRNA* | 2 | 426 | | NA | | NA | | NA | | NA | | NA | | NA | | NA | | NA | | NA | | NA | | NA | |  |
| Multi-analyte | 2 | 887 | | NA | | NA | | NA | | NA | | NA | | NA | | NA | | NA | | NA | | NA | | NA | |  |
| *DNA*-based | 17 | | 2818 | | 89.94 | | <0.01 | | 95.36 | | <0.01 | | 0.64 [0.53,0.74] | | 0.91 [0.82,0.96] | | 7.0 [3.8,12.9] | | 0.39 [0.31,0.51] | | 18 [10,32] | | 0.84 [0.80 - 0.87] | | 0.33 |  |
| *DNA*-based group includes *ctDNA*, methylation and multi-analyte biomarkers.  PLR, positive likelihood ratio; NLR, negative likelihood ratio; DOR, diagnostic odds ratio; AUC, area under the curve; CTC, circulating tumor cell; *ctDNA*, circulating tumor *DNA*. | | | | | | | | | | | | | | | | | | | | | | | | | | |

| **Table S6.** Analysis of concentration of cell-free *DNA* | | | | | | | | | |
| --- | --- | --- | --- | --- | --- | --- | --- | --- | --- |
| Factors | Group | Sample | Mean | SD | Median | Min | Max | P value | K-W test |
| TNM Stage | I-III | 941 | 11.34913 | 13.60541 | 8.64 | 0.12 | 223.07 | 0.00010 | 1-2-3 |
|  | I | 496 | 10.28036 | 14.10443 | 7.575 | 0.12 | 223.07 | 0.000406 | 1-2 |
|  | II | 230 | 12.72409 | 14.27403 | 9.86 | 0.12 | 120.25 | 0.000015 | 1-3 |
|  | III | 215 | 12.34386 | 11.368 | 10.03 | 0.13 | 104.39 | 0.219484 | 2-3 |
| T Stage | T1-4 | 339 | 11.93611 | 18.53443 | 8.5 | 0.12 | 223.07 | 0.00010 | 1-2-3-4 |
|  | T1 | 97 | 9.429897 | 25.23258 | 1.09 | 0.21 | 223.07 | 0.000012 | 1-2 |
|  | T2 | 160 | 12.84713 | 16.71295 | 10.05 | 0.35 | 120.25 | 0.000000 | 1-3 |
|  | T3 | 55 | 13.84291 | 12.17647 | 13.7 | 0.12 | 83.04 | 0.000226 | 1-4 |
|  | T4 | 27 | 11.65704 | 7.910697 | 11.1 | 0.73 | 25.6 | 0.029938 | 2-3 |
| N Stage | N0-2 | 333 | 11.72982 | 17.98579 | 8.5 | 0.12 | 223.07 | 0.00010 | 1-2-3 |
|  | N0 | 201 | 9.524279 | 19.13286 | 1.7 | 0.12 | 223.07 | 0.000004 | 1-2 |
|  | N1 | 72 | 16.63528 | 20.05451 | 11.35 | 0.54 | 120.25 | 0.000001 | 1-3 |
|  | N2 | 60 | 13.23183 | 6.869507 | 12.245 | 0.38 | 34.54 | 0.300171 | 2-3 |
| Age | Total | 853 | 10.8646 | 14.02858 | 7.84 | 0.12 | 223.07 | 0.000000 | 1-2 |
|  | <65 | 468 | 9.274466 | 11.54235 | 6.985 | 0.12 | 120.25 |  |  |
|  | ≥65 | 385 | 12.79753 | 16.36468 | 9.03 | 0.12 | 223.07 |  |  |
| Gender | Total | 853 | 10.8646 | 14.02858 | 7.84 | 0.12 | 223.07 | 0.98403 | 1-2 |
|  | F | 376 | 10.47069 | 11.41097 | 7.83 | 0.13 | 94.23 |  |  |
|  | M | 477 | 11.17509 | 15.7942 | 7.9 | 0.12 | 223.07 |  |  |
| Smoker | Total | 774 | 10.85121 | 10.17802 | 9.03 | 0.12 | 104.39 | 0.000000 | 1-2 |
|  | No | 376 | 8.653723 | 8.703625 | 7.515 | 0.12 | 104.39 |  |  |
|  | Yes | 398 | 12.92724 | 11.01159 | 10.64 | 0.38 | 94.23 |  |  |
| Histopathology | Total | 941 | 11.34913 | 13.60541 | 8.64 | 0.12 | 223.07 | 0.00010 | 1-2-3 |
|  | LUAD | 611 | 12.03435 | 15.30246 | 9.03 | 0.12 | 223.07 | 0.014090 | 1-2 |
|  | LUSC | 183 | 12.1182 | 8.973286 | 10.78 | 0.35 | 52.76 | 0.000000 | 1-3 |
|  | Others | 147 | 7.543605 | 9.785778 | 4.42 | 0.12 | 64.09 | 0.000000 | 2-3 |
| SD, standard deviation; Min, minimum; Max, maximum; K-W test, Kruskal-Wallis test; LUAD, lung adenocarcinoma; LUSC, lung squamous cell carcinoma. | | | | | | | | | |

| **Table S7.** Concordance between *ctDNA* and pathological response to neoadjuvant therapy | | | | | | | | | |
| --- | --- | --- | --- | --- | --- | --- | --- | --- | --- |
|  | *ctDNA* Responder | | | | *ctDNA* Non-responder | | | | Row Total |
| Study | ① | ② | ③ | subtotal | ① | ② | ③ | subtotal |  |
| MPR or pCR | 6 | 8 | 13 | 27 | 0 | 8 | 1 | 9 | 36 |
| Non-MPR or pCR | 1 | 15 | 26 | 42 | 5 | 38 | 46 | 89 | 131 |
| Column Total | 7 | 23 | 39 | 69 | 5 | 46 | 47 | 98 | 167 |
| Response Rate (%) | 86% | 35% | 33% | 39% | 0% | 17% | 2% | 9% |  |
| Study ①: Yue et al., Transl Lung Cancer Res, 2022.  Study ②: Kris et al., Ann Oncol, 2021.  Study ③: Forde PM et al., N Engl J Med, 2022.  *ctDNA* responder in Study ① corresponded to *ctDNA* change of relative delta mean variant allele fraction, while Study ② and ③ represented the *ctDNA* clearance from detectable down to undetectable.  Response rate (%) means the percentage of patients who experienced either MPR or pCR.  *ctDNA*: Circulating tumor *DNA*; MPR: Major pathologic response; pCR: Pathological complete response. | | | | | | | | | |
