# Supplementary material for: Potential clinical utility of liquid biopsy in early-stage non-small cell lung cancer
Source: BMC Med. 2022 Dec 14;20:480. doi: 10.1186/s12916-022-02681-x (PMC9749360; doi:10.1186/s12916-022-02681-x)
Supplement: Supplementary file 3 — Additional file 3: Figure S1. PRISMA flow diagram. Figure S2. Deeks’ funnel plot in diagnostic analysis. Figure S3. Funnel plot in prognostic analysis of RFS at (A) preoperative and (B) postoperative time point; and OS at (C) preoperative and (D) postoperative time point. [file 12916_2022_2681_MOESM3_ESM.docx]

**Figure S1.** PRISMA Flow Diagram

Full-text articles excluded, with reasons
(n = 528):

Advanced stages (n = 74)

Reviews, letters, case reports or comments (n = 56)

Insufficient data (n = 62)

Did not assess relevant outcomes (n = 300)

Other carcinomas (n = 11)

Overlapping patients (n = 10)

Case less than 10 (n = 8)

Did not report in English (n = 4)

Others (n = 3)

Studies included with 2 assess both values

•Diagnostic values (n = 34)

•Prognostic values (n = 21)

Additional records identified through other sources
(n = 20)

Records excluded
(n = 5464)

Records identified through database searching
(n = 8388)

Studies included in qualitative synthesis
(n = 53)

Full-text articles assessed for eligibility
(n = 581)

Records screened
(n =6045)

Records after duplicates removed
(n = 6045)

## Identification

## Eligibility

## Included

## Screening


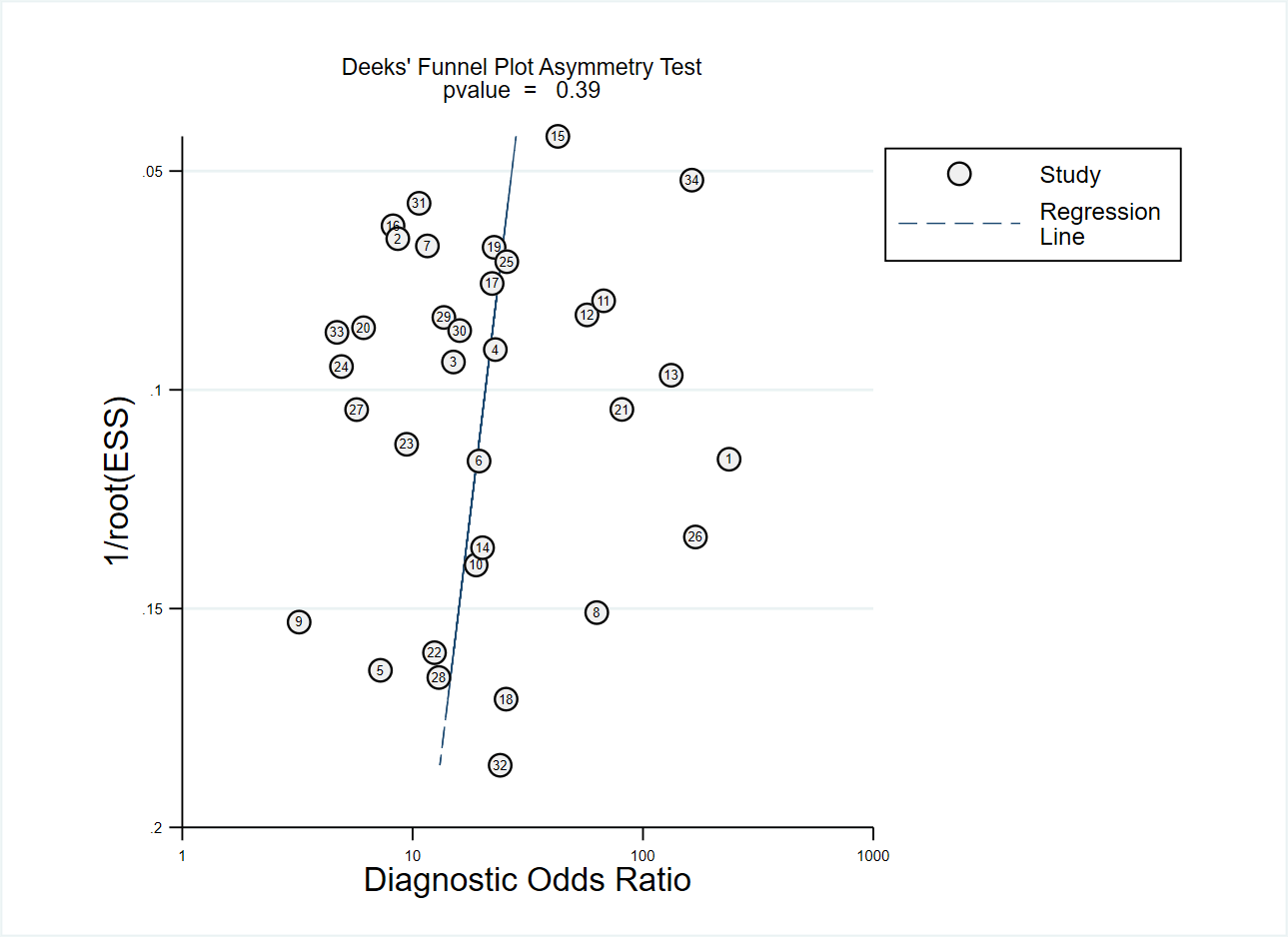


**Figure S2.** Deeks’ Funnel Plot in Diagnostic Analysis

**B**

**A**


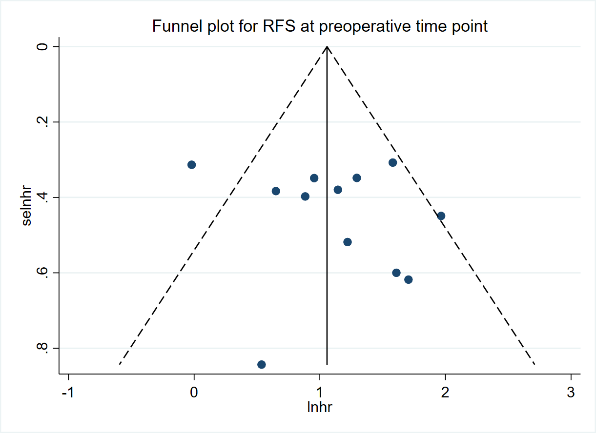

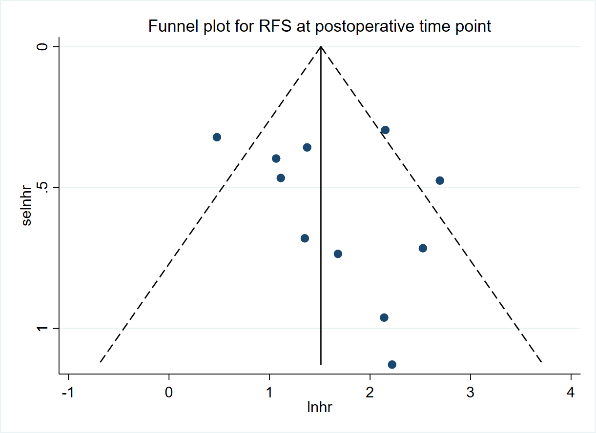


**Egger’s test, P = 0.435**

**Egger’s test, P = 0.407**

**C**

**D**

**
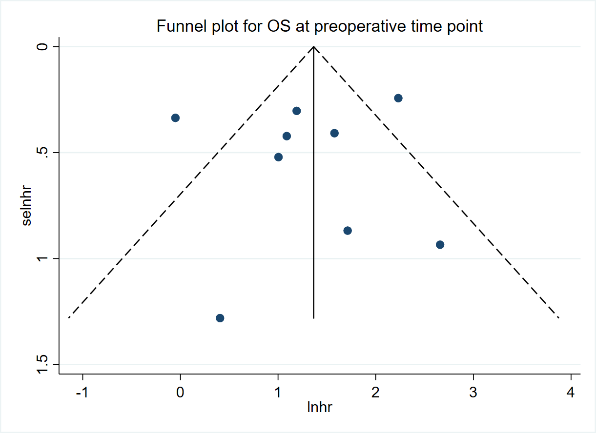

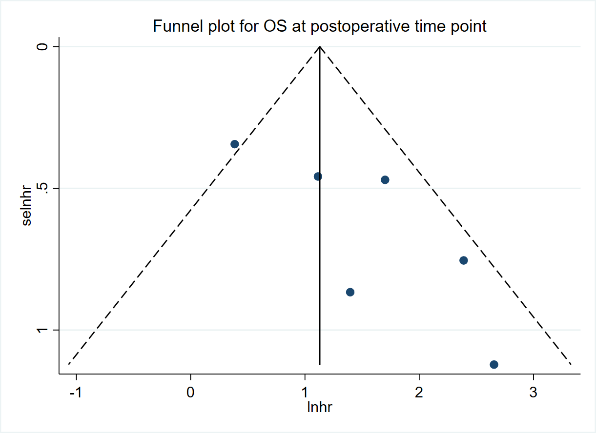
**

**Egger’s test, P = 0.062**

**Egger’s test, P = 0.730**

**Figure S3.** Funnel Plot in Prognostic Analysis of RFS at **(A)** Preoperative and **(B)** Postoperative Time Point; and OS at **(C)** Preoperative and **(D)** Postoperative Time Point
